# Long-term consequences of traumatic experiences: an assessment of former political detainees in romania

**DOI:** 10.1186/1745-0179-1-17

**Published:** 2005-09-26

**Authors:** Dana Bichescu, Maggie Schauer, Evangelia Saleptsi, Adrian Neculau, Thomas Elbert, Frank Neuner

**Affiliations:** 1 Department of Psychology, University of Konstanz, Fach D-25, D-78457 Konstanz, Germany; 2 Department of Psychology, University of Iasi, Blvd. Carol I, Nr. 11, Iasi, 700506 Romania; 3 vivo, Cassela Postale no. 17, Castelplanio Stazione, I-60032 Ancona, Italy

## Abstract

**Background:**

Research has suggested that organized violence and torture have long-term psychological effects that persist throughout the lifespan. The present survey aimed at examining the prevalence of posttraumatic stress disorder (PTSD), and other disorders and symptoms, all present in old age, as long-term consequences of politically motivated violence in a comparison design.

**Methods:**

A group of former political detainees (N = 59, mean age 73.5 years) who had been arrested by the Romanian communist regime were compared to an age- and gender-matched control group (N = 39). PTSD was assessed using a structured clinical interview (CIDI). The investigation of the clinical profile was further accomplished by self-rating measures for anxiety, depression, and health-related functioning, as well as by clinician-administrated interviews for substance abuse, dissociation, and somatization symptoms.

**Results:**

Lifetime prevalence of PTSD was 54%. In the case of participants left untreated, PTSD persisted, often over four decades, such that current PTSD was diagnosed still in a third of the survivors. Other clinical conditions such as somatization, substance abuse, dissociative disorders, and major depression were also common among the former political detainees and often associated with current PTSD.

**Conclusion:**

Our findings suggest that political detention may have long-term psychological consequences that outlast the changes in the political system.

## Background

Research on victims of organized violence and torture has suggested that prolonged severe traumata produce long-term psychological effects that persist even into old age. Many comparative epidemiological studies have been conducted to investigate the long-term consequences of organized violence, but there is still a lack of information regarding psychological and physical status of the survivors in their late life. In particular, there is little information about the consequences of subjection to political persecution in the formerly communist countries of Eastern Europe. The extensive review of empirical support for the co-occurrence of complex symptoms led to the conclusion that this was mainly the consequence of prolonged, repeated trauma that usually occurred during captivity, such as in prisons, concentration camps, or labor camps [1]. It has been argued that traumatized individuals may suffer from various combinations of symptoms over time and that this pattern of symptoms reflects the adaptation to psychological trauma on the cognitive, affective and behavioral levels, particularly when this has occurred early in life [2].

Information about the persistence of posttraumatic sequelae into old age comes mostly from investigations on Holocaust survivors and on POW-s in World War II. Irrespective of the particular type of assessment used to determine mental health consequences, most studies have found that survivors suffered from long-lasting impairment even later in life [3]. In addition, more recent studies that applied the diagnostic criteria for PTSD reported the persistence of posttraumatic symptoms and high rates of related co-morbid disorders in many victims who developed PTSD in the aftermath of the trauma, for 40–50 years after the end of World War II and up into old age [4-10]. Prospective research has also focused on the late effects of combat exposure, showing relatively few PTSD symptoms in survivors [11].

However, these results may be influenced by the participants' selection procedures. Earlier clinical investigations of Holocaust consequences late in life have also underlined an increase in the severity of depressive and somatic symptoms as an outcome of the unsuccessful emotional processing of the trauma [12]. These data have lately been sustained by other studies, which have suggested that late life might be a period of increased vulnerability in the aftermath of severe trauma [10,13].

Research concerning posttraumatic consequences of organized violence has lately focused mainly on refugees. In addition to the very relevant studies on refugees, the importance of research on victims of organized violence who continued to live in their countries of origin has been emphasized [14], as the only way in which the effects of organized violence could be separated from the ones of living in exile. More recently, increasing attention has been paid to psychological disturbances in the aftermath of political imprisonment specific to former East Germany [15-17]. Long-term consequences such as PTSD, anxiety, depression, dissociation and vegetative complaints have been found.

In countries with a history of dictatorship, terror, and institutionalized violence, both political opponents and arbitrary victims fell prey to severe abuse. In Romania, political imprisonment and deportation procedures, commonly used under King Carol's dictatorship and during the Nazi era, were reinforced beginning with August 23, 1944, when Romania started the war against Germany and the Communists came to power under the supervision of the Soviet state which had administrative control over Romania at that time. Whereas the earlier political coercion had been directed against Jews and individual political opponents, under the Communist regime it extended to the whole Romanian society, becoming part of everyday life and generating pervasive fear and terror, which affected social structures as well as individual behaviour [18]. Between 1944 and 1947, most legionary members and political personalities were imprisoned, executed or expatriated. The information regarding detainment forms during this period is scarce and contradictory [19]. On April 13, 1948, a constitution of the Soviet type was adopted. Thousands of people were arrested during the following days, including members of the Communist Party that were not "clean". To liquidate the regime's opponents, the secret police, i.e. a new instrument of repression with unlimited power, was created. The juridical and economic systems were reformed, along with the nationalization of the private properties. Any kind of behaviour that did not meet the rules set by the regime was violently repressed [20], and the Gulag-style system of forced-labor camps and political prisons resulted in a high number of deaths. Beginning with 1949, the imprisonment regime became much harsher: the prisoners were unmercifully beaten, they were not allowed to lie down during the day, and food was very little and bad [19]. Beginning with 1950, the system of forced-labor camps was established. Testimonies say that political dissidents, opponents to the new system, and other arbitrary victims were subjected to extermination practices such as forced labor, starvation, and torture (e.g. [21]). Another characteristic of the Communist system of political detainment was the regular transfer from one prison to another, so as to prevent any type of contact among the detainees, make them loose contact with the outside world, or for administrative reasons, e.g. need of working force in certain labor camps [22,23]. During this period, after further ulterior Communist propaganda, denunciation flourished and numerous sudden changes in political orientation occurred [23]. Since the change of political leadership in 1965, a mitigation of extreme policy stated that the political detention regime be officially ended. The following period, until 1989, was mainly characterized by other means of control, repression and censorship, during which the "memory of terror" ensured the political systems' efficient functioning, and political imprisonment was seldom used, being disguised under the procedures applied to common convicts [22,23].

The present study documents a clinical psychological investigation that was conducted in Romania on a sample of Romanian former political detainees now in their late sixties and seventies. A control group of elderly people that have not undergone political persecution served to explore the cause of the symptoms. Our aims were the following: (1) to explore the severity of traumatic conditions during detention; (2) to investigate the range of PTSD, the health-related functioning, anxiety, depression, substance abuse, somatization, and dissociation within this population in a controlled comparison; (3) to analyze the interrelationships between PTSD diagnosis and the clinical profile of the participants; (4) to compare clinical conditions between the former political prisoners who received psychological support and those who did not.

## Methods

### Participants

The study design included two groups: a political detainee group and a control group. The group of political detainees consisted of N = 59 (1 female) Romanian survivors of the Communist political imprisonment practice and persecution, who had been political activists or just arbitrary victims. Regardless of their political affiliation, time of imprisonment, or actual need for help, participants in this group were Romanian citizens arrested after 1948, according to the decision of the Military Court, some arrest warrant, the administrative order given by the Ministry of Internal Affairs or even without any written order, simply by means of the abuse of the police state organs. All possessed a legal status as former political prisoners, according to documents that prove their imprisonment. Study announcements were made at the Romanian Association of Former Political Detainees (AFDPR) in Iasi, Suceava, Botosani, and Brasov, an anti-Communist organization militating for the rights of the former political prisoners, and at the Medical Rehabilitation Clinic (MRCT) in Iasi, a branch of the IRCT (International Rehabilitation Council for Torture Victims) in Moldavia, which offers medical and psychological support for victims of political persecution and torture. The psychological support sessions were conducted by a psychiatrist at the clinic headquarters, and consisted of a regular group treatment procedure in which the participants were able to share their stressful experiences and to discuss their problems. From the 83 former political detainees (71 male and 12 female) who were approached and invited to participate, after informed consent, 58 (82%) of the men and 1 (8%) of the women agreed to enter the study. We have no explicit information on the reasons for non-participation. According to the employees of the two institutions, many women had been sexually abused during prison and they might not be willing to report such experiences, which is consistent with the analysis of written testimonies on this topic [23]. Twenty-eight of the participants were responders to the announcements at AFDPR and 31 of them responded to the announcements at MRCT. Participating survivors who were recruited from the MRCT received current medical and psychological assistance, as mentioned above. Our sample was representative in terms of age distribution, education and occupational status of the former political prisoners registered at the Association of Former Political Detainees and at the Medical Rehabilitation Clinic.

The control group (N = 39, 3 female) was a general population sample recruited from a city clinic where mostly old age pensioners from Moldavia were under treatment for rheumatoid and arthritic problems. These persons had responded to the hospital's employees' announcements for participants in the research study. From the control group, one participant reported a history of torture and four and a half years of political imprisonment between 1980–1985, because of his origins (his parents had been political opponents arrested during the Communist regime), another reported ordinary conviction to two-and-a-half- month imprisonment due to his refusal to become a member of the Communist party, and a third one reported having been persecuted because he had studied abroad. With these few exceptions, in the other cases of control subjects, their life during the Communist era had had a normal course, without interferences of the state authorities. For matching purposes, exclusion criteria were age and gender.

Overall, the two groups were comparable in terms of institutional social contacts, most socidemographic characteristics, WWII veterans, psychiatric history of the family, former psychiatric illness, and traumatic experiences other than those related to political imprisonment and persecution (Table 1). The mean age of the group of former political detainees was 73.5 years (range 60–87, SD ± 6.9), and the mean age for the control group was 71.4 years (range 59–88, SD ± 7.0). Compared to the control group, former political detainees had reached a higher educational level. Among the traumatic experiences not related to political imprisonment, war-zone exposure, accidents, exposure to natural disasters (e.g., earthquake, storm, flood, fire), witnessing trauma, physical assault, traumatic events involving a close relative or friend were reported. Only one case of sexual abuse was recorded. A significant higher percent of former political detainees in comparison with controls reported physical assault. All participants signed an *informed consent *containing a detailed description of the research purposes. The study was approved by the Konstanz University Ethical Review. The interviews were carried out in July–October 2001 and in August–November 2002 at the subsidiaries of the institutions participants had been recruited from.

**Table 1:** Sociodemographic and other characteristics of former political detainees and control subjects^1^

Variables	Political detainees (n = 59)	Control subjects (n = 39)	p
Gender			n.s.
Male	98% (58)	92% (36)	
Female	2% (1)	8% (3)	
Highest education level			< 0.001
Primary school	0% (0)	8% (3)	
Secondary school	2% (1)	28% (11)	
High school	25% (15)	28% (11)	
Technical college	15% (9)	10% (4)	
University	58% (34)	26% (10)	
Marital status			n.s
Single	3% (2)	0% (0)	
Married	83% (49)	87% (34)	
Divorced	2% (1)	3% (1)	
Widowed	12% (7)	10% (4)	
Occupational status			n.s.
Employed	14% (8)	23% (9)	
Retired	86% (51)	77% (30)	
WWII veterans	17% (10)	18% (7)	n.s.
Psychiatric history of the family	14% (8)	18% (7)	n.s.
Former psychiatric illness	3% (2)	10% (4)	n.s.
Other traumas	3.4 (1.5)	2.8 (1.3)	n.s.
War zone exposure	86% (51)	82% (32)	n.s.
Accident	61% (36)	59% (23)	n.s.
Natural disaster	46% (27)	49% (19)	n.s.
Witnessing injury	31% (18)	23% (9)	n.s.
Sexual abuse	2% (1)	0% (0)	n.s.
Physical assault	59% (35)	21% (18)	< 0.001
Trauma that happened to someone close	56% (33)	44% (17)	n.s.

^1^Frequencies for percentages and standard deviations for means are presented in parentheses

### Instruments and Procedure

An extensive evaluation of the psychological status for all participants was conducted by self-report and diagnostic clinical interview measures. A clinical psychologist and two master-degree students in psychology who had been specifically trained in the use of diagnostic interviews carried out assessments. A translation/back-translation technique concerning contents, criteria, semantic, notional and procedural correspondence was used to develop Romanian versions of the instruments [25], except for the cases in which adapted and validated Romanian versions were available, e.g., Mini Mental State Inventory (MMSI) and Beck Depression Inventory (BDI).

#### The Assisted Self-Report Section

Every subject completed a series of self-report surveys with the assistance of an interviewer. The next sequence for the completion of these items was the following:

(1) Demographic characteristics of the participants were recorded by the interviewer.

(2) Mini Mental State Inventory (MMSI; [26]). Based on the evaluation of mental functioning, subjects presenting substantial cognitive impairment were excluded.

(3) Short Form-12 Health Survey (SF-12; [27]). This questionnaire was used for the assessment of the health-related functioning within daily living. The second item within the questionnaire had to be adjusted to the local living conditions. This item aims at showing to what extent moderate activities such as "moving a table, pushing a vacuum cleaner, bowling or playing golf" are limited by health. The list of activities was modified and became "moving a table, pushing a vacuum cleaner, taking a short walk or riding the bicycle" (as bowling and golf are not typical activities in Romania). We obtained an abbreviated physical and mental health profile consisting of two summary measures describing the health-related quality of life, Physical Component Summary (PCS) and Mental Component Summary (MCS).

(4) State-Trait-Anxiety Inventory (STAI; [28]). In this study, this instrument was used to assess the intensity of anxiety in the present situation, i.e., at the time of the interview, and to determine the current general level of anxiety.

(5) Beck Depression Inventory (BDI; [29]). We used this questionnaire for screening the current co-morbid depression symptoms and for the diagnosis of depression disorders according to DSM-IV criteria.

(6) An adjusted version of the Persecution and Maltreatment Checklist[17,30] This is a semi-structured interview that was developed to assess the traumatic conditions during political imprisonment in Eastern Germany. By using a pre-testing and a reviewing procedure, the instrument was cross-culturally adjusted to the conditions of political imprisonment in Romania. As the development, structure and function of the political imprisonment practice in all Central and South-East European communist regimes has followed the soviet model [24], we considered this instrument to be adequate for our research purposes. Historical research and the review of written testimonies have demonstrated that the system of political detention in Romania was characterized by the use of additional specific procedures [18,19,22]. Consequently, we reviewed the survey by adding new items referring to mock executions, forced labor, torture, forced standing, and blindfolding. Next, we pre-tested the instrument with potential respondents who were survivors of political imprisonment, to make sure that the questions were clear, reasonable, and free of undue burdens. This proved that the items were adequate to their experiences during political detention and revealed the common use of another procedure, confinement in overcrowded cells. Accordingly, we added a new question concerning this procedure. The interview included questions referring to the severity of traumatic experiences during two periods (pre-trial detention and punitive imprisonment). We collected information across several maltreatment categories (solitary confinement; confinement in overcrowded cells; darkroom confinement; special confinement, i.e., arrest; blindfolding; physical maltreatment, e.g., unsystematic beating, torture, forced labor, sleep deprivation, forced standing, exposure to extreme temperatures, starvation, refused medical help; psychological maltreatment, e.g., threats, offenses; witnessing physical torture; witnessing psychological torture; witnessing death; spies among detention colleagues; death threat; mock executions). Each item was rated for the application of a specific maltreatment category (yes or no) and for self-ratings of associated distress on a seven-point scale, ranging from 1 "not at all disturbing" to 7 "extremely disturbing". Scores lower than 4 were regarded indicative of low distress, whereas 4 was considered the cut-off value, indicating a moderate level of distress. Scores ranging from 5 to 7 were considered to indicate a high level of distress. A supplementary open question regarding personal experiences was asked for each category.

#### The Diagnostic Interview Section

Classically structured surveys were used in the assessment for a broad range of psychiatric disorders. Post-traumatic symptoms and co-morbid symptoms were obtained by means of one interview.

(1) PTSD-section of the Composite International Diagnostic Interview (CIDI; [31]). Trained clinical interviewers assessed current PTSD diagnosis according to the DSM-IV criteria. Participants in the clinical group reported imprisonment experiences as most traumatic. Consequently, posttraumatic symptoms in this group were always determined in relation to maltreatment experiences during imprisonment from the event-checklist mentioned above. Taking into account the complexity of prolonged trauma such as repeated or continuous torture, a separate approach of the experiences that had occurred during imprisonment would have been rather artificial. The instrument also assesses lifetime PTSD. Additionally, lifetime traumatic experiences were screened by using the event list mentioned at the beginning of the interview. In order to be sure that the symptom structure was being examined, the CIDI-typical skip rules were not applied; rather, each respondent was asked the full set of questions.

(2) Alcohol and substance abuse/dependence, somatization symptoms, former psychiatric illness, and psychiatric history of the family were evaluated by the corresponding sections of the Anxiety Disorders Interview Schedule for DSM-IV (ADIS-IV; [32]).

(3) Finally, the Structured Clinical Interview for DSM-IV Dissociative Disorders (SCID- D; [33]), was used for the diagnosis and assessment of dissociative symptoms and disorders.

### Data Analysis

Group differences for continuous variables were evaluated by means of Student t test. Group comparisons for categorical variables were calculated by χ^2^-tests. Logistic regression analysis was accomplished to study relationships between education and diagnoses, between education and symptoms, between current PTSD and dissociative disorders, between psychological support and current diagnoses and symptoms.

We also examined the inter-correlations between the PTSD-section of CIDI and the STAI, BDI, and SF-12 scales. To this purpose, the symptom clusters' frequencies from the PTSD-section of CIDI (which reflect, respectively, the symptoms of re-experiencing, avoidance, and arousal) were summed to create an overall frequency of posttraumatic symptoms. Pearson correlation coefficients (r) were performed with this PTSD scale score using the total anxiety scores (STAI scale), combined with the total depression scores (BDI scale) and the PCS and MCS measures (SF-12 scale).

## Results

### The History of Repression and Exposure to Traumatic Conditions during Detention

Forty-eight (81.4%) of the former political detainees reported that they had been subjected to state persecutions a few months before imprisonment: surveillance, sanctions, and interrogations. The most frequent reason for imprisonment was the membership within an anticommunist organization followed by anticommunist propaganda (Table 2). The average age at the time of arrest was 20.0 years (range 16–55, SD ± 9.39). Following arrest, there was a period of remand with an average duration of 4.6 months (range 0–24, SD ± 4.4). This time period consisted of interrogations that usually took place at the closest secret police station from the persons' residence. The pre-trial detention was followed by a formal military trial and the victim was convicted to several years of political prison for allegedly committed crimes against the communist regime.

**Table 2:** The distribution of reasons for imprisonment within the group of former political detainees

	Former political detainees (n = 59)	
	
Reason for imprisonment	n	%
Membership in an organization/political party	19	32.20
Membership in a legionary organization	8	13.56
Rebellion against the Communist system	6	10.17
Anticommunist propaganda	14	23.73
Support of political opponents	2	3.39
Victims of arbitrariness	10	16.95

**Figure 1:**
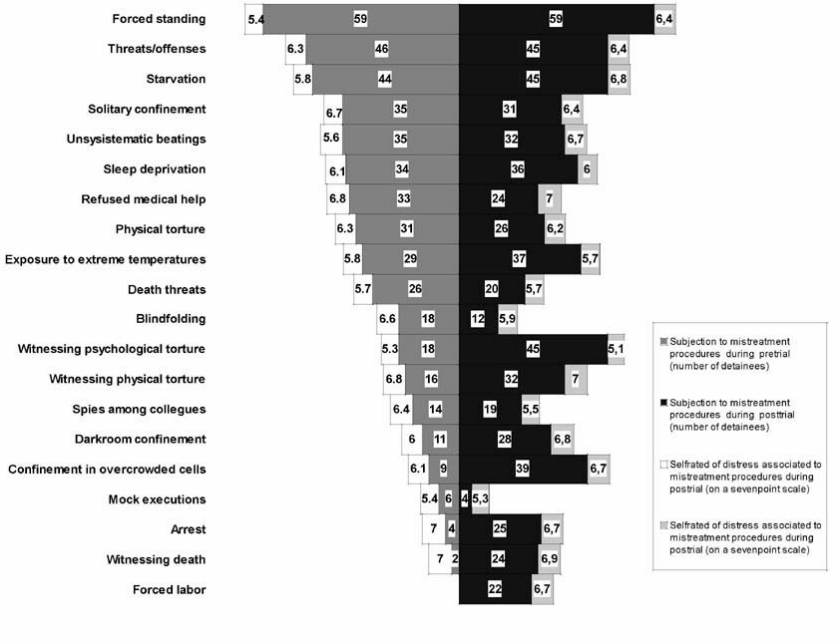
Prevalence of traumatic conditions during detention and associated distress *, as reported by former political detainees (N = 59). * Associated distress was measured on a seven-point scale.

On the average, imprisonment lasted 68.7 months (range 13–198, SD ± 45.7). The imprisonment occurred in 4.0 prisons on the average (range 1–12, SD ± 2.4). Traumatic conditions were more frequently reported during punitive detention (M = 10.3, range 1–18, SD ± 5.0) as compared to pretrial detention (M = 7.8, range 1–15, SD ± 3.6; *t*(58) = 4.7, P < .001). The characteristics of traumatic conditions during pretrial and punitive detention, and the associated level of distress are presented in frequency order in Figure 1. Among the procedures of physical torture, the most common was the falanga, i.e. the beating of the feet' soles (39% during pre-trial detention, respectively 36% during punitive detention). All measures have been associated with high distress (as measured on a seven-point scale). When asked to specify the most stressful aspect of pre-trial detention, 24 respondents (40.7%) reported physical maltreatment, 19 (32.2%) of them reported psychological maltreatment, and 6 (10.3%) of them reported isolation. During punitive detention, 11 respondents (18.6%) reported starvation, 9 (15.3%) of them reported forced labor, and 8 (13.6%) of them reported physical maltreatment as the most stressful aspects of punitive detention.

On the average, 43.9 years (range 14–53, SD ± 7.2) had passed between the time of release from prison and the present investigation. Three of our participants had been imprisoned again in the 80ies, the last of them being released in 1991. Most of the former political detainees (64.4%) reported that they had been subjected to further persecutions after being released from imprisonment, such as professional disadvantages (e.g., forced to work below their level of qualification, not allowed to continue studies and to receive higher degrees), oppression (e.g., forced displacement, surveillance and regular interrogations), and other kinds of discriminations. These post-prison repressive events lasted until the end of 1989, when a rapid political change put an end to the communist regime.

### Disorders, Symptoms and Results on Self-Report Scales

All survivors of political persecution and 62% (N = 18) of the control group reported traumatic events that fulfilled the stressor criterion (A-criterion) for PTSD. Table 3 shows the diagnoses and the self-reported psychological problems separately for former political detainees and for controls. The two groups differed significantly on PTSD and on most of the other clinical indicators. 31% (N = 18) of political detainees fulfilled the diagnostic criteria of current PTSD at the time of the assessment. The lifetime PTSD prevalence in survivors was 54%; no cases of delayed onset of PTSD were recorded. In contrast to controls, somatization symptoms, substance abuse (alcohol and tobacco) and dissociative disorders were also frequently reported by survivors, whereas a current major depression was only significant for one-tailed testing (Table 3). Such a difference was supported by significantly elevated depressions scores (BDI) in survivors of the imprisonment, compared to controls who scored within the normal range. Among somatization symptoms, pain and pseudoneurological symptoms were particularly common. The most frequently diagnosed dissociative disorder among former political detainees was depersonalization disorder (N = 12, 20.3%). Both groups scored high on state and trait anxiety (STAI) as self-rating scales, whereby former political detainees had significantly higher scores than control subjects on state anxiety. Using a logistic regression, there was no significant association between education and any of the clinical conditions. The educational level measured in the number of school years had no covariate effect on the self-report measures except for anxiety rates (state-trait anxiety: F(1,94) = 8.3, p < 0.01, state anxiety: F(1,94) = 4.4, p < 0.05, trait anxiety: (F(1,94) = 11.4, p < 0.001).

**Table 3:** Psychiatric diagnoses and ratings on self-report scales of former political detainees and comparison subjects

	Political detainees (n = 59)		Control subjects (n = 39)		Analysis	
			
Clinical condition	n	%	n	%	χ^2^	p
PTSD, current	18	30.5	1	2.6	11.7	< .001
PTSD, lifetime	32	54.2	3	8.3	22.2	< .001
Major depression (current)	16	27.2	5	12.8	2.9	<.1
Dissociative disorders	20	33.9	4	10.3	7.1	< .01
Somatization symptoms	28	47.5	7	17.9	8.9	< .01
Substance abuse	22	37.2	5	12.8	7.0	< .01

Self-rating scale	Mean	SD	Mean	SD	*t*	p

Beck Depression Inventory	12.5	9.3	8.2	6.7	2.4	< .05
State-Trait Anxiety Inventory	84.1	15.8	78.7	14.9	1.7	n.s.
State anxiety	41.3	10.2	37.0	8.8	2.1	< .05
Trait Anxiety	42.9	7.2	41.7	7.3	0.8	n.s.
PCS (SF-12)	38.7	10.5	36.5	9.7	1.0	n.s.
MCS (SF-12)	46.1	11.9	44.0	12.5	0.8	n.s.

Only two (3%) of the former political detainees had no PTSD symptoms, while the majority (76%) reported more than six symptoms. The most frequent specific symptoms within the group of survivors were recollections of trauma (78%), distress when reminded of trauma (68%), bad dreams or nightmares about the trauma (64%), and avoidance of trauma thoughts (63%). The least common symptoms were restricted affect (19%) and limited expectations concerning the future (22%). The comparison of mean frequencies of current PTSD symptom clusters within the group of former political group pointed towards a significantly higher count of intrusive and avoidance as compared to hyperarousal symptoms [t(58) = 3.3; p < .01; t(58) = 2.3; p < .05].

Within the group of survivors, the PTSD score correlated positively with STAI-anxiety (r = 0.38, p < 0.01), with BDI-depression (r = 0.44, p < 0.001), and negatively with SF-12-physical health (r = -0.42, p = 0.001). Also, the depression score (BDI) was positively correlated with STAI-anxiety (r = 0.36, p < 0.01) and negatively correlated with SF-12-mental health (r = -0.34, p = 0.01). Current PTSD associated with dissociative disorders (OR (2.4, 28.3) = 8.3, p < 0.001).

Although there was no significant difference regarding lifetime PTSD between these two groups, a significantly higher prevalence of current PTSD was noted among former political detainees who did not receive psychological support (Table 4). Dissociative disorders and substance abuse were also more frequently diagnosed among those who had not received psychological assistance. Logistic regression analyses showed that psychological support was predictive of current PTSD diagnosis (OR (0.1, 0.5) = 0.3, p < 0.05), of dissociative disorders (OR (0.1, 0.9) = 0.3, p < 0.05), and of substance abuse (OR (0.1, 0.8) = 0.2, p < 0.05).

**Table 4:** Differences between former political detainees who have and who have not had psychological support

Diagnoses	Psychological support (n = 31)		No psychological support (n = 28)		Analysis	
		
	n	%	n	%	χ^2^	p
**PTSD, current**	5	**16**	13	**46**	-6.4	< .01
PTSD, lifetime	17	55	15	54	0.1	n.s.
**Dissociative disorders**	7	**23**	13	**46**	-3.7	< .05
Somatisation symptoms	14	45	14	50	-0.6	n.s.
**Substance abuse**	8	**26**	14	**50**	-3.7	< .05
Major depression	9	29	7	25	0.1	n.s.

Self-rating scale	Mean	SD	Mean	SD	t	p

Depression (BDI)	10.3	5.7	14.8	11.8	1.9	n.s.
Anxiety (STAI)	84.8	17.0	83.5	14.6	1.4	n.s.
PCS (SF-12)	39.9	9.6	37.2	11.3	1.0	n.s.
MCS (SF-12)	45.9	12.3	46.4	11.6	-0.2	n.s.

## Discussion

The present study is based on the clinical assessment of a sample of 59 survivors of the political detention regime in Romania in their late life, and it aimed at exploring long-term psychological consequences of traumatic conditions during detention within this group. Participants in our sample were young or very young at the time of arrest. As compared to other studies on political victims of former communist regimes [15,17], our results show that the exposure to traumatic conditions within the Romanian sample was more severe in terms of subjection to traumatic conditions (exposure to a mean of 8.6 forms of maltreatment) and detention duration (5 years and 9 months on average). Traumatic conditions such as forced standing, threats and offences, starvation followed by solitary confinement, unsystematic beatings, and sleep deprivation were very frequent during both pre-trial and punitive detention. During punitive detention, these traumatic experiences were commonly accompanied by confinement in overcrowded cells, work in labor camps, exposure to extreme temperatures, and torture witnessing. In addition, political detainees were very often moved from one prison to the other. These data suggest that the Romanian political detention regime was an system based mainly on methods of gradual physical and psychological weakening, which was also demonstrated by means of historical analysis [22]. This conclusion is also consistent with the political detainees' accounts of their most disturbing experiences.

The investigation of posttraumatic symptoms in our study was related to the experience of political imprisonment. Our results add evidence to the qualification of political detention among traumatic consequences with complex long-term consequences [1]. The rate of current PTSD among participants was 31%, whereas the lifetime PTSD prevalence was evaluated at 54%, confirming the hypothesis that PTSD can be a long-term consequence of political detention late in life. These results are similar to those of research on political victims of former Eastern Germany [15,17], as well as to those of studies dealing with psychological effects of the POW experiences in World War II and of the Holocaust after 40–50 years [4,10]. However, in contrast with the studies on former political prisoners from East Germany, most of the participants within the present group of survivors consisted of political activists or people who had expressed their anticommunist beliefs, in one way or another. Although less common, others were arbitrary victims. Consequently, we could not prove the notion of a low rate of PTSD among victims of political imprisonment and torture who had been political activists [14,34]; further persecutions after the release from prison may have contributed to the maintenance of the disorder.

Somatization symptoms (48%) were frequently diagnosed among former political detainees. The prevalence of other disorders such as substance abuse (37%), dissociative disorders (34%) and major depression (27%) was also high. High associations between the current PTSD diagnosis and the prevalence of dissociative disorders confirm their co-morbidity with PTSD, which has previously been found in aged survivors as well [11,35]. Also, PTSD score related to depression, supporting findings on Holocaust survivors [8], and to anxiety and physical health scores. The association of PTSD with most of the other clinical conditions in our survivor group validated our assumption that political imprisonment leads to chronic PTSD and other long-term consequences. Other possible contributors to the prevalence of these co-morbid symptoms and disorders are persistent persecution after release, and lack of rehabilitation and of social support. The high co-morbidity supports the notion of long-term complex severe psychological effects of prolonged, repeated trauma, and indicates that a current PTSD diagnosis would not capture the severe psychological harm present in such cases [1,2]. Moreover, this result is consistent with the studies conducted after a long post-trauma time interval on Holocaust survivors in old age, which have indicated that persistent posttraumatic symptoms may contribute to additional psychological and physical disturbances [10,13].

Some of our participants received psychological support within a clinical context. This group had the same life-time prevalence of PTSD as those left untreated (54%), but exhibited a significant lower rate of current PTSD, substance abuse, and dissociative disorders. Further analyses showed that a lack of psychological support was associated with current PTSD, dissociative disorders and substance abuse. As the assignment to these two groups was not done at random, we cannot ascertain a causal relationship. Nevertheless, as compared to recovery from PTSD in the treated group, which reached a worth-mentioning 70%, spontaneous remission in the untreated sample was very low (less than 15%), suggesting that, once it has developed, PTSD is likely to persist over the whole lifespan. However, treatment seemed not to produce a reduction on the level of intrusion, somatization, and major depression. Possibly, psychological support was efficient only in the reduction of posttraumatic hyperarousal and avoidance, substance abuse, and dissociation. Alternatively, it might be that those who recovered from PTSD sought treatment for their remaining mental health problems, i.e., intrusions, somatization and the collateral depressive symptoms. Given that intrusions and somatization both result from implicit memories that are not sufficiently integrated with explicit autobiographical recollection [36], a trauma-focused treatment would probably be needed [37].

### Limitations of the Study

Recruitment strategies may not have allowed for the estimation of the epidemiological prevalence. First, the participants recruited from one institution did not look for treatment, whereas the other ones had already been offered psychological assistance. However, other recruitment strategies without an institutional frame would have not allowed for the selection of a sufficient number of participants, since most victims are still extremely suspicious when asked to report their experiences [38]. Secondly, 82% of the men and only 8% of the women identified as survivors agreed to participate. The scarce participation of women in this study may explain why the present sample is representative of former political detainees from the Moldavian region of Romania in terms of age distribution, education and occupational status, but not of gender distribution. If highly traumatizing experience such as sexual violence or persistent high anxiety levels precluded subjects from participation in the study, the actual PTSD rate may thus be even higher. In addition, it may well be possible that, in a country with a 75-years life expectancy, a higher fraction of former detainees with severe mental disorders died by the age of seventy, as opposed to those less affected by trauma [39].

The educational level of control subjects was lower than that of survivors. This difference was predictable, since a remarkably high educational level was a distinctive feature of the political detainees population, given that the bourgeoisie and the intelligentsia were the favorite target groups for political persecutions during the former Communist regimes [24]. The covariate effects of education showed that this factor explained the similarity of the two groups with respect to the high levels of anxiety. In addition, hospitalization of controls for rheumatoid and arthritic problems may explain the lack of a difference with respect to physical problems between the two groups. The two groups also differed with respect to exposure to physical assault. The unusual high number of such experiences reported among former political detainees might be an aspect of their subjection to political violence, as suspected by most of them, although they had no proof of it. Yet, none of the participants rated experiences of physical assault as the most traumatic experience. However, the inclusion of a culturally and sociodemographically equivalent comparison group helps validating our findings.

We also acknowledge that the long period of time of 43.9 years on the average, which had passed since the trauma until the moment of the interview, may have affected the validity of the data collected retrospectively with the Persecution and Maltreatment Checklist. On the other hand, the traumatic experiences, reported by the respondents involve "hot" memory, i.e., episodes with strong emotional and sensory components that seem to be highly consistent and long-lasting [36,40].

## Conclusion

The present study shows that PTSD and other mental disorders developed significantly in former political detainees of the Romanian communist regime as a consequence of trauma, maltreatment and other stress factors that acted during the political imprisonment. Across four decades, the disorder persisted in severe forms, particularly in the former political detainees who did not receive psychological assistance. Thus, political detention and post-detention life conditions are likely to have negative long-term effects on the mental health status of former political detainees that may last even into the latest stages of life when left untreated. A high prevalence of co-morbid disorders and symptoms, including somatic complaints, add to the suffering of these survivors. Obviously, these victims need psychotherapeutic help and care that includes rigorous research concerning political persecution. However, in view of a highest efficiency, public recognition and political rehabilitation would also be necessary requirements. Further research on survivors of political persecution in the former Communist countries of Eastern Europe may add helpful data in understanding the psychological effects of this phenomenon.

## Competing interests

The author(s) declare that they have no competing interests.

## Authors' contributions

DB conducted the study in Romania, performed the statistical analysis, and drafted the manuscript. MS designed the study and coordinated the studies activities in Romania. ES participated in the analyses. AN participated in the design of the study and its coordination. TE designed the study and revised the manuscript. FN designed the study and revised the manuscript. All authors read and approved the final version.
